# A distributed data processing scheme based on Hadoop for synchrotron radiation experiments

**DOI:** 10.1107/S1600577524002637

**Published:** 2024-04-24

**Authors:** Ding Zhang, Ze-Yi Dai, Xue-Ping Sun, Xue-Ting Wu, Hui Li, Lin Tang, Jian-Hua He

**Affiliations:** a The Institute for Advanced Studies, Wuhan University, Wuhan 430072, People’s Republic of China; Paul Scherrer Institute, Switzerland

**Keywords:** big data, Apache Hadoop, distributed data processing, microservice architecture

## Abstract

With the development of synchrotron radiation sources and high-frame-rate detectors, the amount of experimental data collected at synchrotron radiation beamlines has increased exponentially. As a result, data processing for synchrotron radiation experiments has entered the era of big data. It is becoming increasingly important for beamlines to have the capability to process large-scale data in parallel to keep up with the rapid growth of data. Currently, there is no set of data processing solutions based on the big data technology framework for beamlines. Apache Hadoop is a widely used distributed system architecture for solving the problem of massive data storage and computation. This paper presents a set of distributed data processing schemes for beamlines with experimental data using Hadoop. The Hadoop Distributed File System is utilized as the distributed file storage system, and Hadoop YARN serves as the resource scheduler for the distributed computing cluster. A distributed data processing pipeline that can carry out massively parallel computation is designed and developed using Hadoop Spark. The entire data processing platform adopts a distributed microservice architecture, which makes the system easy to expand, reduces module coupling and improves reliability.

## Abstract 2

A set of distributed data processing schemes for beamlines with experimental data using Hadoop are presented.

## 1. Introduction

Synchrotron radiation facilities are some of the most powerful tools in science and technology research. After over half a century of development, the diffraction-limited storage ring has become the most dominant trend, *i.e.* fourth-generation light source. MAX IV (Tavares *et al.*, 2016 ▸) in Sweden, ESRF-EBS (Revol *et al.*, 2021 ▸) in France and Sirius (Liu *et al.*, 2021 ▸) in Brazil are the first three constructed fourth-generation light sources in the world, and there are more than ten laboratories that plan to build a new facility or upgrade their recent third-generation facilities (Li *et al.*, 2022 ▸). In mainland China, HEPS (Jiao *et al.*, 2019 ▸) in Beijing and HALS (Yang *et al.*, 2019 ▸) in Hefei are under construction. The Wuhan Advanced Light Source (WALS) phase I project is designed as a fourth-generation light source (Li *et al.*, 2021 ▸), consisting of a low-energy storage ring (1.5 GeV), a LINAC working as a full-energy injector and ten beamlines. Based on the hybrid-7BA lattice structure, the low-energy storage ring reaches the soft X-ray diffraction limit. By using a 3.5 T superB magnet, the photon energy of the storage ring is extended to the hard X-ray region. The fourth-generation light sources will exceed the performance of previous sources by one or more orders of magnitude in terms of the important parameters such as brightness, coherence and shortness of pulse duration (Grabowski *et al.*, 2021 ▸). The high degree of coherence will allow efficient focusing of the synchrotron beams to the nanometre range. It will allow an effective application of coherence-based techniques such as coherent diffraction imaging potentially reaching sub-nanometre resolution. It will also extend photon correlation techniques into the regime of nanoseconds (Khubbutdinov *et al.*, 2019 ▸). The gain in coherent flux brought by the fourth-generation light sources allows experimental data to be obtained within a few seconds (Westfahl *et al.*, 2018 ▸). Along with the development of the light source, high-frame-rate detectors are also widely used in synchrotron radiation experiments on beamlines. Recent advances in the fourth-generation synchrotron light sources and innovative detector technology (*e.g.* higher acquisition rates and larger area-detector dimensions) (Pithan *et al.*, 2023 ▸) lead to generating massive amounts of data in users’ synchrotron radiation experimental processes, and beamlines need to have the ability to perform large-scale parallel data processing to ensure that the processing speed of experimental data matches the speed of data growth (Khan *et al.*, 2018 ▸). At the same time, a massive amount of data also provides a solid foundation for big data technologies, such as machine learning. The application of machine learning is expected to be a significant trend in the development of data analysis technology for beamlines (Hill *et al.*, 2020 ▸). There are a few beamline stations that have applied machine learning algorithms to accelerate data analysis (Vollmar *et al.*, 2020 ▸), and have even directly used machine learning algorithms for autonomous experiments, greatly reducing human intervention to pursue experimental efficiency (Noack *et al.*, 2021 ▸). Data processing on the beamline has entered the era of big data. High performance computing (HPC), implemented with the message passing interface (MPI) protocol (Walker & Dongarra, 1996 ▸), developed and matured before the advent of the big data era. As a result, the beamline typically relies on HPC for parallel data processing. MPI is a parallel computing framework based on message passing, which involves numerous communication intricacies in the program, making implementation challenging. With the increasing scale of computing, the proportion of communication time also grows, resulting in decreased program efficiency. MapReduce (Dean & Ghemawat, 2008 ▸) in big data technology is a programming model for parallel computing of large-scale datasets. It is easy to program, highly scalable, and capable of solving ultra-large-scale parallel computing problems efficiently. At the same time, it is highly reliable and has a strong fault tolerance ability. Therefore, MapReduce will be more advantageous when applied to large-scale data-parallel computing. NSLS-II has developed an extension module that enables MPI support in the distributed big data processing engine Spark. This allows HPC applications based on MPI to be executed on Spark computing engines (Malitsky, 2016 ▸). However, there are no set of beamline distributed data parallel processing schemes based entirely on the big data technology framework.

Hadoop is an open-source Apache project for developing software for reliable, scalable, distributed computing (He & Lai, 2018 ▸; Zhang *et al.*, 2017 ▸). The Apache Hadoop software library is a framework that enables the distributed processing of large datasets across clusters of computers using simple programming models. It is designed to scale up from single servers to thousands of machines, each offering local computation and storage. Hadoop is widely chosen as the preferred framework for distributed big data solutions in companies or organizations because of its high fault tolerance, reliability, efficiency and low cost. This paper presents a distributed processing scheme for beamline experimental data based on the Hadoop big data architecture. It utilizes Hadoop HDFS and HBase as distributed file storage systems and databases, and adopts Hadoop YARN as the resource scheduler for the distributed computing cluster. Additionally, a distributed data automatic processing pipeline is designed and developed using Hadoop Spark. In detail, the paper is organized as follows: Section 2 provides an overview of the system architecture; Section 3 introduces the construction of Hadoop clusters, as well as the design, development and testing of distributed data processing pipelines; Section 4 introduces the development of the application layer based on the microservice architecture; and finally Section 5 summarizes the entire paper.

## 2. Architecture

Hadoop, as an underlying transparent architecture, has three core components (Ma *et al.*, 2023 ▸):

*HDFS (Hadoop Distributed File System)* is a distributed file system that serves as the foundation for data storage and management in the Hadoop system. It is a highly fault-tolerant system that can detect and respond to hardware failures. HDFS simplifies the consistency model of files and provides high-throughput application data access, making it suitable for applications with large datasets.

*YARN (Yet Another Resource Negotiator)* is a new resource manager for Hadoop. It is a universal resource management system that provides unified resource management and scheduling for upper-level applications.

*MapReduce* is a programming framework for distributed computing programs. It serves as the core framework for users to develop Hadoop-based data analysis applications. The main purpose of MapReduce is to combine user code and default components into a comprehensive distributed computing program that can be executed simultaneously on a Hadoop cluster.

In addition, Hadoop uses the Hadoop Common module, which provides fundamental support for the three above components.

With the widespread application of Hadoop technology, Hadoop has developed into an enormous ecosystem with many related components. In addition to the basic core components of Hadoop, they include the Distributed Coordination Service – Zookeeper; the Distributed Column-Storage Database – HBase; the Workflow Scheduling System – Oozie; and the Data Warehouse – Hive. Moreover, there are various distributed computing architectures, such as the data batch processing framework Spark and the data stream processing framework Storm (Ravichandran, 2017 ▸; Zhi *et al.*, 2022 ▸; Islam *et al.*, 2012 ▸; Thusoo *et al.*, 2009 ▸; Zaharia *et al.*, 2012 ▸; Cha & Wachowicz, 2015 ▸).

**Figure 1:**
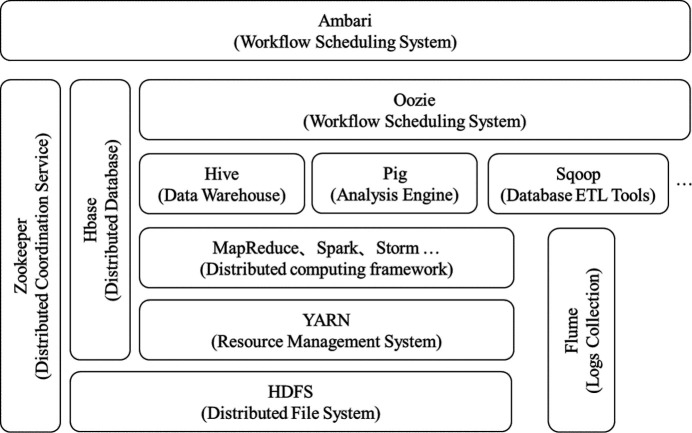
Hadoop ecology.

This paper utilizes the Hadoop ecosystem (Fig. 1 ▸) to build a distributed storage, management and processing big data platform for the experimental data of synchrotron radiation biological macromolecular crystallography. HDFS is used to build a distributed file storage system for experimental crystallography data. Distributed computing for the automatic processing of crystallographic data is achieved by combining the Hadoop distributed batch processing framework Spark and cluster resource management system YARN. Finally, the processed results are saved to the distributed database HBase. The traditional monolithic application architecture cannot flexibly respond to the increasing business needs of big data platforms. In this case, the microservice architecture model can be adopted, which divides traditional monolithic applications into a group of small services based on the concept of divide and conquer. These services can communicate, coordinate and cooperate with each other (Song *et al.*, 2018 ▸).

**Figure 2:**
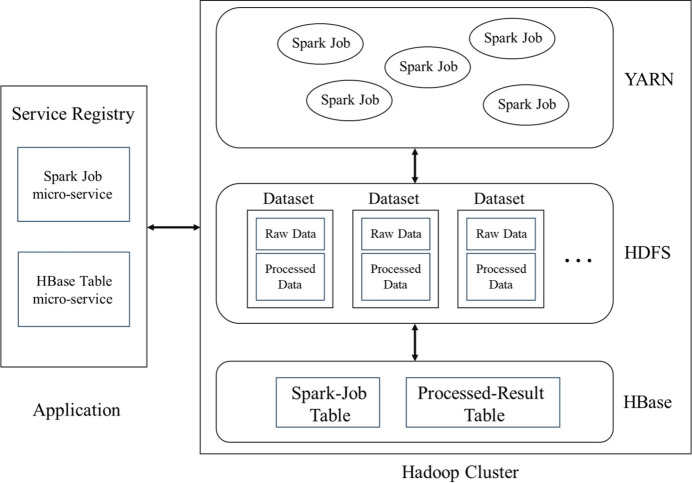
Architecture of the data processing platform.

The entire system architecture can be divided into a Hadoop-based data storage/computing layer and a microservice architecture application layer, as shown in Fig. 2 ▸. In Hadoop clusters, distributed automatic processing jobs in YARN read experimental raw data from HDFS for calculation, and the result files and intermediate files generated during the process are stored in HDFS. HBase extracts the processing results from the result files and stores them in the corresponding tables in the database. Information related to data processing jobs is also stored in the database. In the application layer, there are primarily microservices related to Spark distributed automatic processing jobs and HBase data table operations.

## 3. Distributed data processing

### 3.1. Construction of a Hadoop distributed cluster

In terms of software, HDFS, YARN and HBase in the Hadoop ecosystem are chosen to build a distributed processing cluster in this paper.

HDFS is an important component used for massive data storage and management in the Hadoop project; it is a distributed file storage system based on Google’s GFS (Ghemawat *et al.*, 2003 ▸). HDFS is a typical master/slave architecture distributed system, where an HDFS cluster consists of a NameNode and some DataNodes (Wang *et al.*, 2012 ▸). The NameNode is mainly used to manage metadata (file name, size, storage location, *etc*.) and process file access requests, while the DataNode is specifically used for data storage. Meanwhile, a SecondaryNameNode is added to the cluster, which will generally be in the standby state. When a NameNode fails, the SecondaryNameNode will take over its role as the NameNode.

YARN (Liu *et al.*, 2016 ▸; Yao *et al.*, 2021 ▸) is a resource management component proposed by Hadoop version 2.0. The Hadoop YARN system consists of multiple work nodes and resources, which are managed by a centralized ResourceManager and multiple distributed NodeManagers. The ResourceManager owns all resource allocation decisions in the system and is responsible for resource allocation of all applications in the cluster. The NodeManager manages independent compute nodes in the Hadoop cluster and is mainly responsible for communicating with the ResourceManager.

HBase is an open-source implementation of Google Bigtable (Chang *et al.*, 2008 ▸), which utilizes HDFS as a file storage system and Zookeeper as a collaborative service. In the HBase cluster, the HBase Master coordinates multiple RegionServers, detects the state of each RegionServer, and balances the load between RegionServers. The RegionServer manages the tables and implements read and write operations. The client connects directly to the RegionServer and communicates with it to obtain data from HBase.

In terms of hardware, the distributed cluster is built using the infrastructure provided by the Advanced Light Source Research Center of Wuhan University. The version of Hadoop used is 3.3.4, and the version of HBase is 2.5.4. Two CentOS7.6 servers are used to build a two-node cluster, and the deployment of the Hadoop components is shown in Table 1 ▸.

The two nodes are named Hadoop-node1 and Hadoop-node2. The directory structure in HDFS is almost identical to the local file directory structure. Here, there are *raw_data* and *processed* subfolders under the single crystallographic data directory, where the *raw_data* folder stores the experimental raw data of the crystals, such as HDF5 files, and the *processed* folder stores the intermediate files and processed result files generated by the automatic processing pipeline. The resource information of the cluster is given in Table 2 ▸.

### 3.2. Distributed automatic processing pipeline for crystallography

#### 3.2.1. Pipeline design

The design of the Hadoop Map­Reduce framework is limited to the concept of Map and Reduce, and it cannot adapt to the computation of all types of data. As a result, various computing frameworks have emerged to address the issue of processing different types of data. Spark is one such framework.

**Figure 3:**
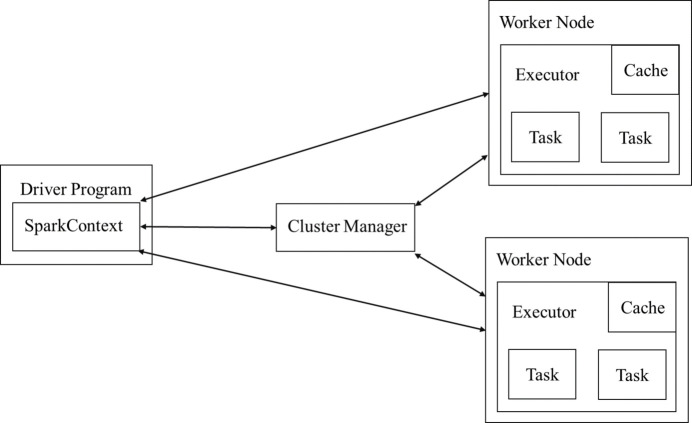
Architecture of Hadoop Spark.

Spark is a dominant distributed batch computing framework in big data computing. It supports various types of functionality, including offline batch processing, SQL-like processing, machine learning, graph computation, and more. The Spark architecture consists of a Cluster Manager and multiple Worker nodes, as shown in Fig. 3 ▸. The Cluster Manager is responsible for controlling the entire cluster and monitoring Workers. The Driver is the main process that executes the Spark Application, which contains the SparkContext corresponding to the application. A Worker node is responsible for controlling computation and starts and manages an Executor or Driver. An Executor is a process that runs in a Worker node for a specific application.

When constructing the runtime environment for the application, a SparkContext instance will be initialized. SparkContext requests the running resources of the Executor from the Cluster Manager. SparkContext builds the execution code into a DAG (Directed Acyclic Graph), which is decomposed into several stages. Each stage is composed of a TaskSet, which is then assigned to the requested Executor for execution on a Spark Task basis. The Spark application is capable of multi-node distributed parallel computing (Liao *et al.*, 2018 ▸; You *et al.*, 2023 ▸). Spark abstracts data into an RDD (Resilient Distributed Dataset), and all data processing in Spark is based on RDD operations. The RDD is essentially a collection of data, which is further divided into several partitions (Li *et al.*, 2023 ▸). A Spark Task is responsible for processing an RDD in a single partition, so the number of Spark Tasks running in distributed parallel is determined by the number of RDD partitions.

The DIALS (Diffraction Integration for Advanced Light Sources) project (DIALS, 2023 ▸) is a collaboration between Diamond Light Source, Lawrence Berkeley National Laboratory and CCP4 to develop a new software suite for the analysis of crystallographic X-ray diffraction data. The core aim of DIALS is to allow the development of a wide range of algorithms within a single framework. The workflow of DIALS is decomposed into a number of discrete tasks, including spot finding, indexing, refinement, integration, *etc*. These tasks exchange information via data files. This decomposition also makes testing of the DIALS software more straightforward and facilitates its inclusion within automated data-analysis systems (Winter *et al.*, 2018 ▸). After processing a set of crystal diffraction data through this sequence of tasks, information such as the cell parameters, space group, completeness and resolution of the crystal can be obtained. In the entire DIALS data processing workflow, the two steps of spot-finding and integration involve multi-process parallel processing of crystallographic data. This paper utilizes Spark to transform spot-finding and integration in DIALS into tasks that can perform multi-node parallel computing within a computer cluster, and then integrates them with other tasks in DIALS, following the order of the DIALS workflow. Thus, a distributed Spark-DIALS pipeline capable of automatically processing crystallographic data is designed. The key to the transformation of the original DIALS is to convert the parallel tasks into Spark Tasks so that the tasks can be distributed to multiple nodes in the cluster for distributed computing. The design of the Spark Task needs to confirm the partitioning of the single-partition RDD and the corresponding RDD operation. This paper references the design of single-process data processing in the original DIALS spot-finding and integration source code. The single-process processing in the original DIALS converts the data to be processed into a data collection and then performs a traversal operation on the data collection. This data collection can be directly regarded as an RDD in Spark, and we only need to partition it (which also partitions the Spark Task). By encapsulating the operations of data collection in the original DIALS loop into a unified processing function, the function can be seen as an operation on a single-partition RDD in each Spark Task. After passing this function into the Spark RDD *map* operator, the single-process traversal operation is converted into a distributed computing Spark Task. Taking the original DIALS integration as an example, a Manager class specifically for managing parallel tasks is designed in the original DIALS. The Manager can complete the partitioning of parallel tasks and generate a task list. In the original DIALS, the code for executing parallel tasks in a single-process loop is as follows:[Chem scheme1] 
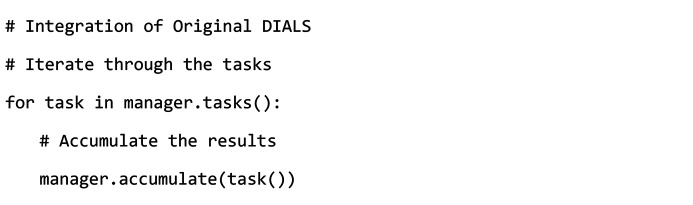
 In each iteration, a single task is retrieved from the list of tasks in the instantiated manager object. The accumulate method of the manager object is then called within the loop to accumulate the results of the calculation for each task. The original DIALS is implemented in Python. The modification of the original DIALS integration using PySpark (PySpark, 2024 ▸) is as follows: 
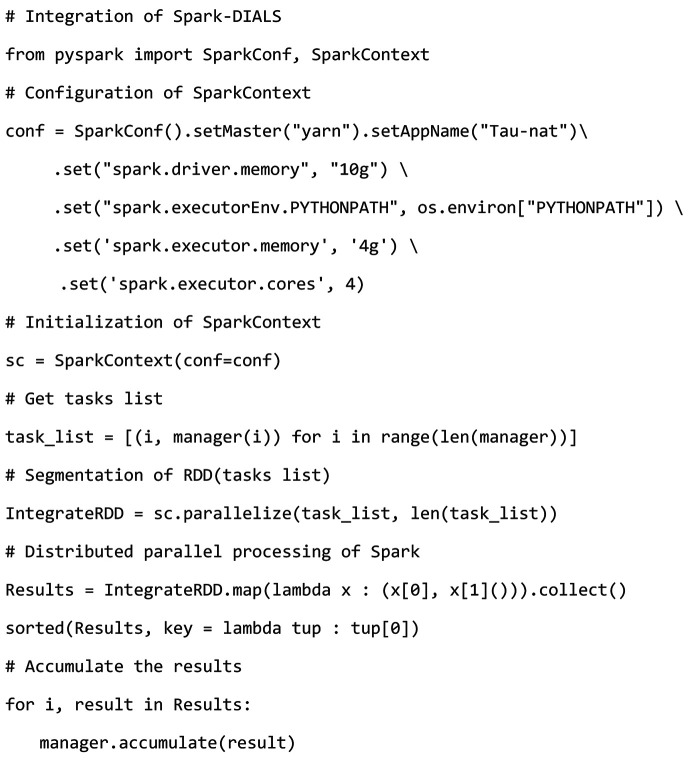
 Parallel tasks distributed to the cluster nodes may disrupt the original order of the tasks. Therefore, the original list of tasks is converted to a list of tuples, where each tuple contains the task serial number. This list can be treated as an RDD, which is a collection type of data. To partition the tuple list, the SparkContext instantiated object *sc* calls the parallelize method. After partitioning, IntegrateRDD calls the *map* operator, which passes in an anonymous lambda function that defines operations on a single binary tuple in the RDD. This lambda function returns a binary tuple containing the task number and the calculation result of the corresponding task. IntegrateRDD then calls the *collect* operator to gather the calculation results from all partitions. Finally, a loop is used to aggregate all the results into the manager.

**Figure 4:**
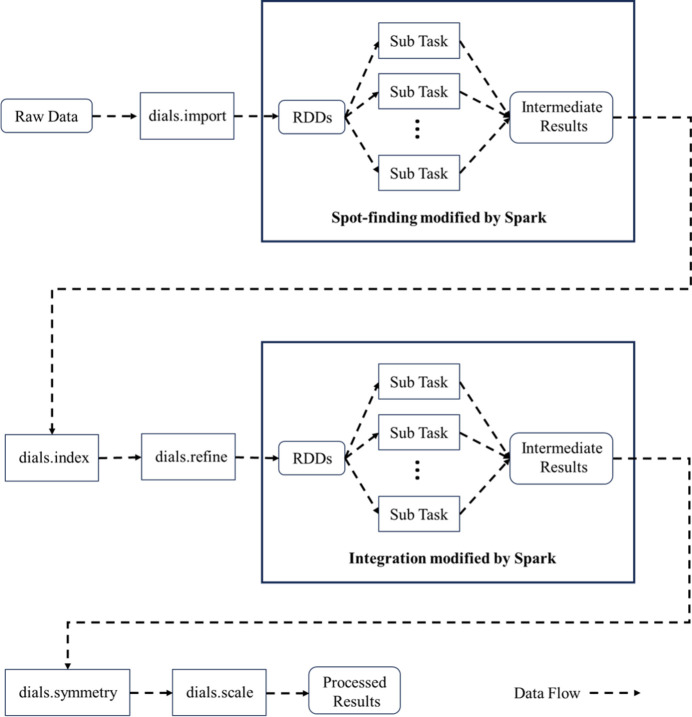
Workflow of Spark-DIALS pipeline.

After the completion of each task in the original DIALS, the result in memory is saved as a file. Subsequent tasks will then read the result file into memory for further operations. In the design of Spark-DIALS, the reading and writing of files between independent tasks is omitted. Tasks will directly retrieve the results generated by their upstream tasks from memory to continue processing. This connects each independent task in DIALS into a continuous data processing pipeline. At present, the Spark-DIALS pipeline primarily incorporates dials.import, dials.find_spots, dials.index, dials.refine, dials.integrate, dials.symmetry and dials.scale from the original DIALS. The entire pipeline workflow is shown in Fig. 4 ▸.

#### 3.2.2. Pipeline testing

The raw experimental data used for pipeline testing were an example dataset consisting of 1800 thaumatin diffraction patterns collected at the BioMAX beamline, MAX IV (Finke & Nan, 2022 ▸). The size of the entire dataset is 5.15 GB. The crystals of thaumatin were grown from solutions of thaumatin (20–50 mg ml^−1^) in NaK tartrate (1.0 *M*), HEPES pH 7 (100 µ*M*) and 25% glycerol. The experimental acquisition parameters for this dataset are shown in Table 3 ▸.

**Figure 5:**
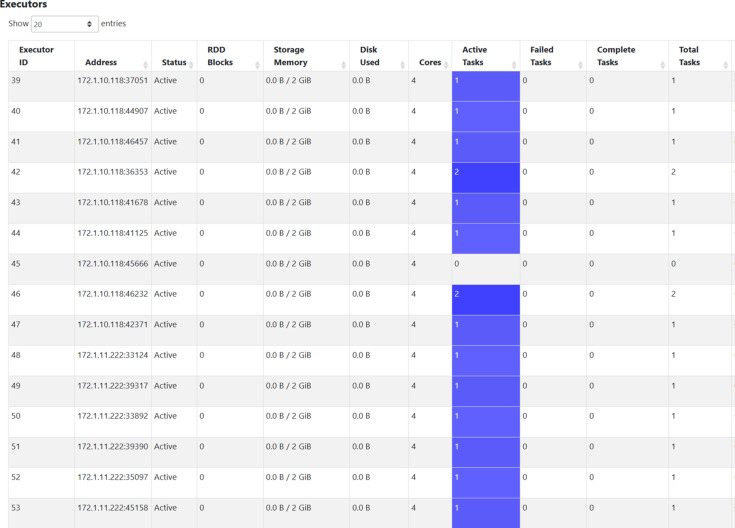
Executor status in the integration phase of the Spark-DIALS pipeline.

The version of Spark used was 3.3.1, and the version of DIALS used was 3.8. First, the Spark-DIALS pipeline job was submitted to Hadoop YARN, and its multi-node distributed computing functionality was verified. After a Spark distributed pipeline job is submitted to the Hadoop YARN cluster, the YARN ResourceManager acts as the Cluster Manager for Spark. This allows the Spark job to run smoothly on the YARN cluster. Fig. 5 ▸ shows the running status of some of the Executors in the cluster obtained from the HistoryServer of Spark at a certain time during the DIALS integration phase of the Spark-DIALS pipeline job. In the built Hadoop cluster, the IP address of Hadoop-node1 is 172.1.10.118, and the IP address of Hadoop-node2 is 172.1.11.222. It can be seen that there are active Spark Tasks on both nodes simultaneously, confirming that Spark-DIALS can indeed distribute the divided parallel tasks to different nodes for distributed computing.

Additionally, the Spark Driver in Spark-DIALS first uses the Spark RDD *map* operator to distribute parallel tasks to each Executor. All task calculations are completed, and the Spark Driver collects all results using the Spark RDD *collect* operator. Spark provides a comprehensive data serialization mechanism for transmitting data between the Driver and the Executor, ensuring the accuracy and completeness of the data. At the same time, the data processing environment configured in each node in the cluster is identical. Therefore, the data processing results obtained by the Spark-DIALS pipeline are the same as those of the original DIALS. *xia2* (Xia2, 2024 ▸) is an expert system for macromolecular crystallography data reduction. It can utilize DIALS as a pipeline to complete the data reduction process automatically. The test compared the results of the dataset processed by xia2(dials) and Spark-DIALS. The analysis results obtained by the two treatments were very similar, both determining that the space group of the crystal is *P*4_1_2_1_2. Other diffraction indices are shown in Table 4 ▸.

**Figure 6:**
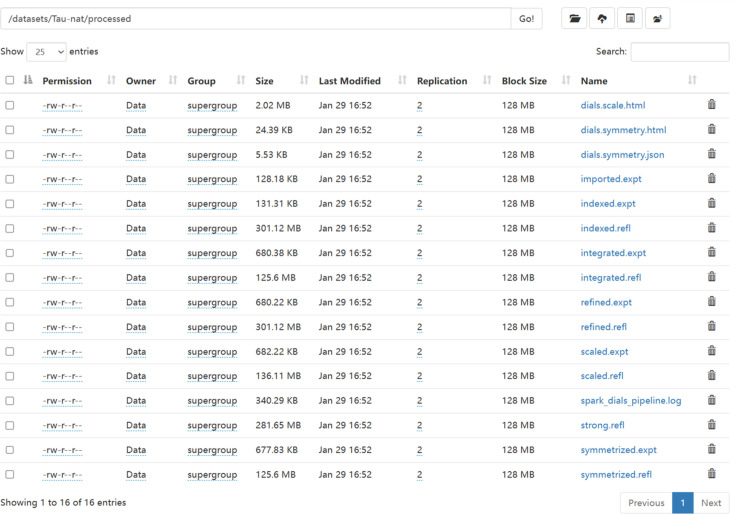
The *processed* folder in HDFS stores distributed automated processing result files.

As shown in Fig. 6 ▸, the data files acquired through the Spark-DIALS pipeline are stored in the *processed* folder in HDFS. This folder contains the result files generated by each task of the original DIALS. Each independent task of the original DIALS generates a log file containing the processing results. Spark-DIALS outputs the contents of these files to the running log file of the entire pipeline (spark_dials_pipeline.log).

Next, we compared the computational efficiency of Spark-DIALS and the original DIALS. In addition to being submitted to the computing cluster for running, Spark jobs can be submitted to a single machine in local mode for parallel processing of data. The running times of spot-finding and integration in Spark-DIALS (Local), Spark-DIALS (YARN) and the original DIALS were measured simultaneously, as shown in Table 5 ▸.

In total, four sets of computational efficiency tests were conducted using 30, 60, 90 and 120 CPU cores. Test data indicate that when 30 CPU cores are invoked, Spark-DIALS performs more than 1.5 times faster than the original DIALS on the spot-finding task, and more than 2.5 times faster on the integration task. The performance of Spark-DIALS is even better in the other three test sets. Spark-DIALS processes the spot-finding task more than twice as fast as the original DIALS and the integration task more than three times as fast. It is evident that the larger the computational volume, the better the efficiency performance of Spark-DIALS. The computational workload of the integration task is significantly larger than that of the spot-finding task. Therefore, the enhancement in computational efficiency of Spark-DIALS is much more pronounced in the parallel processing of integration. In addition, when Spark-DIALS pipeline jobs are submitted to YARN, the information transfer between nodes takes some time when the Spark Driver distributes parallel tasks and collects calculation results. Therefore, the overall computational efficiency of Spark-DIALS (YARN) is lower than that of Spark-DIALS (Local). However, the impact of information transfer on the overall computational efficiency is not significant, and this effect can be eliminated by increasing the network bandwidth between cluster nodes. We compared the overall operational efficiency of the Spark-DIALS pipeline and xia2(dials). It took xia2(dials) 13 min to process the dataset, while Spark-DIALS took 5.5 min to process it when 90 CPU cores were invoked.

The test results indicate that Spark-DIALS, a distributed data processing pipeline for crystallography modified with Spark, is capable of parallel processing on a single machine as well as multi-node distributed computation on a Hadoop cluster. Spark-DIALS offers a substantial improvement in computational efficiency compared with the original DIALS on processing large diffraction datasets. This enhancement enables Spark-DIALS to fully utilize the resources of distributed computing clusters for parallel processing of large-scale experimental datasets. Benefiting from Spark’s serialization mechanism, the computational results of Spark-DIALS are consistent with those of the original DIALS.

## 4. Microservice application

### 4.1. Distributed database

Microservices are autonomous, and a very important feature of autonomy is independent deployment. The modification and deployment of one service should not affect other services. Therefore, in the design of microservice databases, the basic principle that each microservice has a separate database will also be followed.

**Figure 7:**
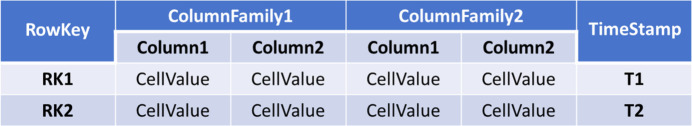
Schematic diagram of the HBase data table structure.

HBase stores data in the form of tables, as shown in Fig. 7 ▸. The table consists of rows and columns, and columns are divided into several column families. Each row of a table has RowKey as the primary key to retrieve the records in the table. Each column in an HBase table belongs to a column family, which must be defined when the table is created. Column names are prefixed with the column family. The real data are stored in a unique unit cell determined by a row key and a column name index. Each cell in HBase holds different versions of the same data, which are distinguished by timestamps.

**Figure 8:**
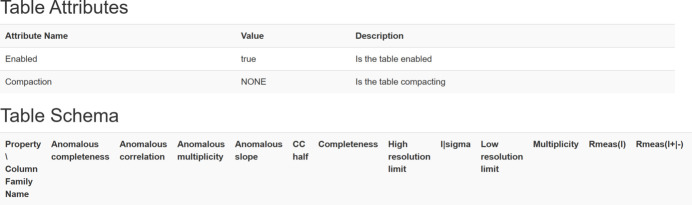
Design of the column families of the data processing result data table.

In this paper, the entire microservice application consists of the Spark pipeline job module and data processing result module, and the corresponding data tables need to be created in HBase for both modules. In HDFS, a single dataset name is used as the file name of the top-level directory, and the dataset name is used as the RowKey in HBase. After each pipeline job is submitted, the corresponding job information and job execution status information need to be saved. The pipeline job data table in HBase creates the column families *job_info* and *status_info*. The unique ID number assigned by Spark to each submitted pipeline job is saved in *job_info*, and the status information of the job submission and execution is saved in *status_info*. After the data processing, information on the cell parameters, space group, resolution and other diffraction indices of the crystals is obtained. The data table of the data processing results will use the obtained diffraction indices as column families, and each column family will store the results of the corresponding index, as shown in Fig. 8 ▸.

### 4.2. Microservice development and registration

This paper uses the high-performance lightweight framework FastAPI (FastAPI, 2023 ▸) to develop the microservice module functionality into a RESTful API. In the Spark pipeline job module, two APIs, for job submission and job query, are provided. The route of the job submission API needs to include the dataset name as a path parameter. After sending an HTTP PUT request to this API, the backend will submit the Spark job of the corresponding dataset to the cluster and then wait for Spark’s HistoryServer to provide the job ID and job submission status (success/failure) to the frontend and save the results in the pipeline job data table. The route of the job status query API also requires the dataset name to be provided as a path parameter. After sending an HTTP GET request to this API, the backend will first go to HBase to obtain the status of the corresponding job submission and execution in the pipeline job data table. If there are no results in the database, the backend will access Spark’s HistoryServer to query the job status based on the ID number saved in the data table. The results will be returned to the frontend in JSON format and saved in HBase. The returned data format is as follows: 
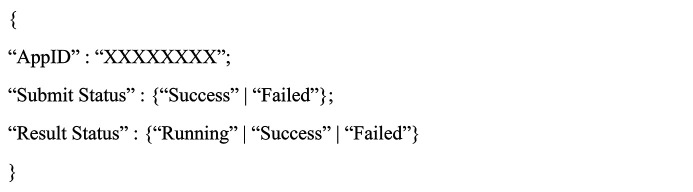


The data processing result module only provides one API. After sending an HTTP PUT request to the API, the backend will extract the processing results from the log file (spark_dials_pipeline.log described previously) in the corresponding dataset folder of the HDFS into the processing result data table in HBase. After sending an HTTP GET request to the API, the backend will obtain all the results from HBase and return them to the frontend in JSON format.

In the microservice architecture, each microservice of the system is deployed on different servers, and the system needs to maintain and manage the instance information of each service through a registry. The registry consists of two parts: the server and the client. The server maintains the information of the services registered to itself while providing interfaces for the services to obtain the information of other services. The client registers its information on the server, making it easy for other services to discover it. It obtains the information of the other services on which it relies and completes interservice invocation.

**Figure 9:**
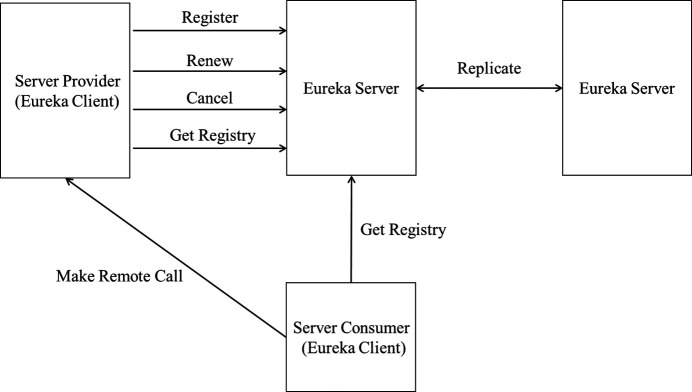
Architecture of Spring Cloud Netflix Eureka.

**Figure 10:**
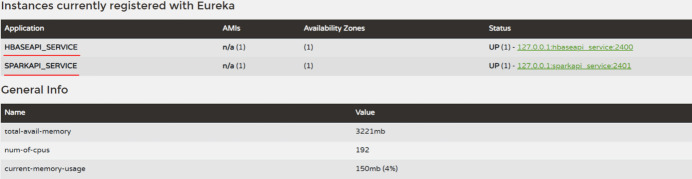
WEB UI of the Eureka Server.

Spring Cloud Netflix Eureka (Eureka, 2023 ▸) is a basic component provided by Spring Cloud for service discovery and registration. Eureka adopts a C/S (Client/Server) architecture, as shown in Fig. 9 ▸. Eureka includes two major components: a Eureka Server and a Eureka Client. The Eureka Server is a service registration center mainly used to provide service registration functions. It maintains a list of available services and stores information about all available services registered with the Eureka Server. The Eureka Client is a microservice system that includes various microservices, which can be divided into service providers and service consumers. The Eureka Server is deployed in a cluster manner, with multiple Eureka Servers synchronously replicating the information of each node in the microservice cluster. The service provider provides services to other nodes in the microservice cluster, and the service consumer initiates remote calls by obtaining the registry information of the Eureka Server. After the Eureka Client completes registration, the service registration center will display information about the microservice applications. As shown in Fig. 10 ▸, the two microservices in this paper can each obtain a unique application name in the registry center – *sparkapi_service* and *hbaseapi_service*. After the microservice completes registration, the Eureka Client can invoke the microservice directly through the application name.

**Figure 11:**
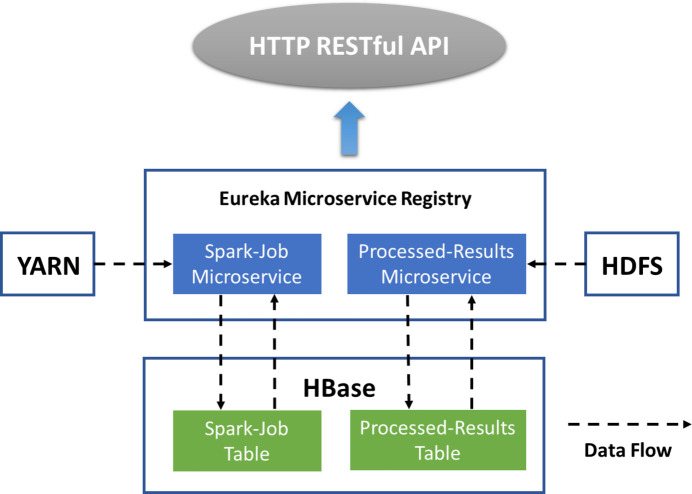
Architecture of application layer.

The entire application layer structure is shown in Fig. 11 ▸. Each microservice in the application layer functions as an independent module, with its own business logic and data that do not interfere with one another. Each microservice can operate independently and autonomously without affecting the operation of other services, ensuring high system reliability. A microservice focuses solely on a specific business function, and its amount of code is small, making the development and maintenance of a single microservice relatively simple. The high cohesion and low coupling characteristics of the microservice architecture make it easy to develop and modify the functional modules in the application, and the entire application is highly scalable.

## 5. Conclusion

The data processing of beamlines has entered the era of big data. In response to the current situation, where there is no large-scale data parallel processing solution based on big data technology, this paper presents a case study on synchrotron radiation biomolecular crystallography to illustrate a beamline distributed data processing scheme based on the Hadoop ecosystem. In this paper, we build a distributed file storage system for experimental crystallography data based on Hadoop HDFS. Additionally, we develop a resource scheduling system for the cluster using Hadoop YARN. Furthermore, we design and develop a distributed automated data processing pipeline (Spark-DIALS) for crystallography by combining Hadoop Spark and DIALS. The results obtained from the Spark-DIALS pipeline are similar to those obtained from the original DIALS. Additionally, Spark-DIALS is not limited to parallel data processing on a single machine and it can perform distributed parallel computing across nodes within a computing cluster as well. The processing efficiency of Spark-DIALS on large datasets is significantly improved compared with the original DIALS, reducing the need for high single machine performance. Compared with the original DIALS, Spark-DIALS has enhanced functionality and performance, enabling it to effectively utilize the resources of all nodes in the computing cluster, thereby solving parallel computing problems with large-scale data. Moreover, the solution utilizes FastAPI and Spring Cloud Netflix Eureka to deploy each functional module in a distributed microservice architecture. In Hadoop HBase, data tables are designed for each module to ensure the autonomy of microservices, thereby improving the flexibility and reliability of the entire system.

Synchrotron radiation light sources are a comprehensive research platform for multiple disciplines. Data processing for the beamline requires not only CPU computing resources but also GPU and FPGA resources, among others. In particular, with the introduction of machine learning technologies such as deep learning, GPUs have a significant advantage in parallel computing for image processing compared with CPUs. The integration of Hadoop distributed big data computing frameworks, such as Spark, with GPU clusters will be a direction for future research.

## 6. Data availability

The data that support the findings of this study are openly available in GitHub at https://github.com/carbee24/Spark-dials/tree/master.

## Figures and Tables

**Table 1:** Deployment of Hadoop components

Node name	HDFS	YARN	HBase
Hadoop-node1	NameNode,	ResourceManager,	RegionServer
	DataNode	NodeManager	
Hadoop-node2	SecondaryNameNode,	NodeManager	Master,
	DataNode		RegionServer

**Table 2:** Resource information of the Hadoop cluster

Cluster resource	Information
CPU	108 vCores (YARN†), Intel Xeon Platinum 9242 at 2.3 GHz
Memory	∼286 GB (YARN†)
Storage	∼2 TB

† 1 YARN vCore = 2 physical cores.

**Table 3:** Data collection parameters of the dataset

Wavelength	0.9763 Å (12.7 keV)
Beamline	BioMAX, MAX IV Laboratory
Detector	DECTRIS EIGER 16M
File format	HDF5 in EIGER Nexus format
Number of frames	1800
Oscillation per frame	0.1° (180° total)
Resolution at detector corners	2 Å
Estimated flux	1.68 × 10^11^ photons s^−1^

**Table 4:** Comparison of analysis results

Diffraction index	Pipeline	Overall	Low	High
High resolution limit	xia2(dials)	1.47	4.00	1.47
	Spark-dials	1.47	4.00	1.47
Low resolution limit	xia2(dials)	54.15	54.18	1.50
	Spark-dials	150.84	151.63	1.50
Completeness	xia2(dials)	83.6	100.0	10.3
	Spark-dials	83.7	100.0	9.3
Multiplicity	xia2(dials)	9.3	12.1	1.2
	Spark-dials	9.2	11.9	1.2
I/sigma	xia2(dials)	9.2	30.4	0.2
	Spark-dials	9.5	28.6	0.3
Rmerge(I)	xia2(dials)	0.124	0.082	0.660
	Spark-dials	0.123	0.094	0.536
Rmerge(I+/−)	xia2(dials)	0.121	0.081	0.456
	Spark-dials	0.121	0.093	0.410
Rmeas(I)	xia2(dials)	0.129	0.085	0.892
	Spark-dials	0.129	0.098	0.735
Rmeas(I+/−)	xia2(dials)	0.131	0.087	0.640
	Spark-dials	0.131	0.101	0.579
Rpim(I)	xia2(dials)	0.037	0.024	0.595
	Spark-dials	0.037	0.028	0.500
Rpim(I+/−)	xia2(dials)	0.050	0.032	0.450
	Spark-dials	0.050	0.038	0.410
CC half	xia2(dials)	0.997	0.996	0.422
	Spark-dials	0.995	0.996	0.518
Anomalous completeness	xia2(dials)	77.1	100.0	0.8
	Spark-dials	77.2	100.0	0.7
Anomalous multiplicity	xia2(dials)	5.1	7.2	1.1
	Spark-dials	5.1	7.1	1.1
Anomalous correlation	xia2(dials)	−0.363	−0.448	0.000
	Spark-dials	−0.437	−0.621	0.000
Anomalous slope	xia2(dials)	0.471		
	Spark-dials	0.513		
dF/F	xia2(dials)	0.071		
	Spark-dials	0.065		
dI/s(dI)	xia2(dials)	0.538		
	Spark-dials	0.509		
Total observations	xia2(dials)	345680	30482	269
	Spark-dials	342370	30028	245
Total unique	xia2(dials)	37358	2514	224
	Spark-dials	37316	2516	203

**Table 5:** Running time of spot-finding and integration

Job type	Find_Spots time (s)	Integration time (s)	CPU cores
Original DIALS	135	322	30
Spark-dials (Local)	86	116	
Spark-dials (YARN)	90	125	

Original DIALS	126	367	60
Spark-dials (Local)	63	107	
Spark-dials (YARN)	63	111	

Original DIALS	123	366	90
Spark-dials (Local)	56	115	
Spark-dials (YARN)	62	114	

Original DIALS	126	346	120
Spark-dials (Local)	57	115	
Spark-dials (YARN)	57	115	
